# Five new additions to the genus *Spathaspora* (Saccharomycetales, Debaryomycetaceae) from southwest China

**DOI:** 10.3897/mycokeys.75.57192

**Published:** 2020-11-09

**Authors:** Shi-Long Lv, Chun-Yue Chai, Yun Wang, Zhen-Li Yan, Feng-Li Hui

**Affiliations:** 1 School of Life Science and Technology, Nanyang Normal University, Nanyang 473061, China Nanyang Normal University Nanyang China; 2 State Key Laboratory of Motor Vehicle Biofuel Technology, Henan Tianguan Enterprise Group Co. Ltd., Nanyang 473000, China Henan Tianguan Enterprise Group Nanyang China

**Keywords:** Five new species, Debaryomycetaceae, Saccharomycetales, yeast taxonomy, d-xylose-fermenting yeast

## Abstract

*Spathaspora* is an important genus of d-xylose-fermenting yeasts that are poorly studied in China. During recent yeast collections in Yunnan Province in China, 13 isolates of *Spathaspora* were obtained from rotting wood and all represent undescribed taxa. Based on morphological and phylogenetic analyses (ITS and nuc 28S), five new species are proposed: *Spathaspora elongata*, *Sp. mengyangensis*, *Sp. jiuxiensis*, *Sp. parajiuxiensis* and *Sp. rosae*. Our results indicate a high species diversity of *Spathaspora* waiting to be discovered in rotting wood from tropical and subtropical southwest China. In addition, the two *Candida* species, *C. jeffriesii* and *C. materiae*, which are members of the *Spathaspora* clade based on phylogeny, are transferred to *Spathaspora* as new combinations.

## Introduction

*Spathaspora* N.H. Nguyen, S.O. Suh & M. Blackw (2006) (Saccharomycetales, Debaryomycetaceae) was introduced, based on a single species, *Spathaspora passalidarum*, which was isolated from a passalid beetle in Louisiana, USA (Nguyen et al. 2006). This species produces asci containing elongate ascospores with curved ends, a unique trait of this genus (Nguyen et al. 2006; Nguyen et al. 2011). Subsequently, *Spathaspora arborariae*, *Sp. boniae*, *Sp. brasiliensis*, *Sp. girioi*, *Sp. gorwiae*, *Sp. hagerdaliae*, *Sp. piracicabensis*, *Sp. roraimanensis*, *Sp. suhii* and *Sp. xylofermentans* were introduced. These were from rotting wood (Cadete et al. 2009, 2013; Lopes et al. 2016; Morais et al. 2017; Varize et al. 2018) and *Sp. allomyrinae* from insects (Wang et al. 2016). *Spathaspora* has been shown to be a polyphyletic group, containing members placed throughout the larger *Spathaspora*/*Candida albicans*/*Lodderomyces* clade of Debaryomycetaceae (Morais et al. 2017; Varize et al. 2018). Several *Candida* species, such as *C. blackwellae*, *C. jeffriesii*, *C. lyxosophila*, *C. materiae*, *C. parablackwellae* and *C. subhashii*, are closely related to *Spathaspora*, based on a phylogenetic analysis of the D1/D2 domain of the nuclear 28S rDNA (nuc 28S) sequences (Cadete et al. 2013; Daniel et al. 2014; Morais et al. 2017; Varize et al. 2018).

Most species of *Spathaspora*, including *Sp. arborariae*, *Sp. brasiliensis*, *Sp. passalidarum*, *Sp. roraimanensis*, *Sp. suhii* and *Sp. xylofermentans*, are economically important because of their ability to ferment d-xylose, the second most abundant sugar in lignocellulosic feedstocks (Nguyen et al. 2011; Cadete et al. 2013; Lopes et al. 2016; Morais et al. 2017). These xylose-fermenting species can be used directly for ethanol production or may provide a source of genes, enzymes and/or sugar transporters to engineer industrial strains for the efficient production of bioethanol from renewable biomass (Wohlbach et al. 2011; Cadete et al. 2013).

*Spathaspora* species are associated with rotting-wood substrates and the insects that occupy this ecological niche (Cadete et al. 2009, 2013; Nguyen et al. 2011; Lopes et al. 2016; Wang et al. 2016; Morais et al. 2017; Varize et al. 2018). They can be found in tropical, subtropical and temperate regions on different continents, but most species are presently known from Brazilian regions (Cadete et al. 2009, 2013; Lopes et al. 2016; Morais et al. 2017; Varize et al. 2018). In China, the genus is under-explored with only three published reports of the species *Sp. allomyrinae*, *Sp. gorwiae* and *Sp. passalidarum* (Ren et al. 2013; Wang et al. 2016). Here, we describe five new species of *Spathaspora* discovered in tropical and subtropical areas of southwest China, based on their morphological characters and molecular phylogenetic analyses.

## Materials and methods

### Sample collection and isolation

Rotting wood samples were collected in two areas of Yunnan Province, China. The areas are located in the Xishuangbanna Primeval Forest Park of Jinghong (21°98'N, 100°88'E) and Jiuxi Mountain Forest Park of Honghe (24°40'N, 103°68'E). The predominant vegetation is characterised as a tropical and subtropical forest biome. The climate is hot and humid, with annual precipitation between 1,100 to 1,600 mm and an average temperature that ranges from 17.2 to 26.4 °C. Sixty decayed wood samples were collected, thirty from each area, during July to August in 2016–2018. The samples were stored in sterile plastic bags and transported under refrigeration to the laboratory over a period of no more than 24 h. The yeast strains were isolated from rotting wood samples in accordance with the methods described by Lopes et al. (2016). Each sample (1 g) was added to 20 ml sterile d-xylose medium (yeast nitrogen base 0.67%, d-xylose 0.5%, chloramphenicol 0.02%, pH 5.0 ± 0.2) in a 150 ml Erlenmeyer flask and then cultured for 3–10 days on a rotary shaker. Subsequently, 0.1 ml aliquots of the enrichment culture and appropriate decimal dilutions were spread on d-xylose agar plates and then incubated at 25 °C for 3–4 days. Different yeast colony morphotypes were then isolated by repeated plating on yeast extract-malt extract (YM) agar (1% glucose, 0.5% peptone, 0.3% yeast extract and 0.3% malt extract, pH 5.0 ± 0.2) and stored on YM agar slants at 4 °C or in 15% glycerol at –80 °C.

### Morphological, physiological and biochemical studies

Morphological and physiological properties were determined according to Kurtzman et al. (2011). Induction of the sexual stage was tested by incubating single or mixed cultures of each of the two strains on cornmeal (CM) agar, 5% malt extract (ME) agar, dilute (1:9 and 1:19) V8 agar or yeast carbon base plus 0.01% ammonium sulphate (YCBAS) agar at 25 °C for 2 months (Cadete et al. 2013; Lopes et al. 2016). Assimilation of carbon and nitrogen compounds and growth requirements were tested at 25 °C. The effects of temperature from 25–40 °C were examined in liquid culture and on agar plates. Ethanol was determined with alcohol oxidase (Sangon Biotech, China) and peroxidase (Sangon Biotech, China), as described previously (Alves et al. 2007).

### DNA extraction, PCR amplification and nucleotide sequencing

Genomic DNA was extracted from the yeasts using the Ezup Column Yeast Genomic DNA Purification Kit, according to the manufacturer’s protocol (Sangon Biotech, China). The nuc rDNA ITS1-5.8S-ITS2 (ITS) region was amplified using the primer pair ITS1/ITS4 (White et al. 1990). The D1/D2 domain of the nuc 28S rDNA was amplified using the primer pair NL1/NL4 (Kurtzman and Robnett 1998). The following thermal profile was used to amplify the ITS and nuc 28S regions: an initial denaturation step of 2 min at 95 °C, followed by 35 cycles of 30 s at 95 °C, 30 s at 51 °C and 40 s at 72 °C, with a final extension of 10 min at 72 °C (Liu et al. 2016). PCR products were directly purified and sequenced by Sangon Biotech Inc. (Shanghai, China). We determined the identity and accuracy of the newly-obtained sequences by comparing them to sequences in GenBank and assembled them using BioEdit (Hall 1999). Newly-obtained sequences were then submitted to GenBank (https://www.ncbi.nlm.nih.gov/genbank/; Table 1).

### Phylogenetic analyses

The sequences obtained from this study and the reference sequences downloaded from GenBank (Table 1) were aligned using MAFFT v. 6 (Katoh and Toh 2010) and manually edited using MEGA v. 7 (Kumar et al. 2016). The best-fit nucleotide substitution models for each gene were selected using jModelTest v2.1.7 (Darriba et al. 2012), according to the Akaike Information Criterion.

Phylogenetic analyses of the combined gene regions (ITS and nuc 28S) were performed using the Maximum Likelihood (ML) and Bayesian Inference (BI) methods. *Candida argentea*CBS 12358 was chosen as the outgroup. ML analysis was performed using MEGA v7 with the GTR+I+G model (Nei and Kumar 2000) and 1,000 rapid bootstrap replicates to estimate branch confidence. BI analysis was performed using a Markov Chain Monte Carlo (MCMC) algorithm in MrBayes v. 3.0b4 (Ronquist and Huelsenbeck 2003). Two MCMC chains, started from random trees for 1,000,000 generations and trees, were sampled every 100^th^ generation, resulting in a total of 10,000 trees. The first 25% of the trees were discarded as burn-in of each analysis. Branches with significant Bayesian Posterior Probabilities (BPP) were estimated in the remaining 7,500 trees. The phylogenetic trees from the ML and BI analyses were displayed using Mega 7 and FigTree v1.4.3 (Rambaut 2016), respectively.

**Table 1.** DNA sequences used in the molecular phylogenetic analysis. Entries in bold are newly generated in this study.

Species	Strain	ITS	D1/D2
*Candida albicans*	NRRL Y-12983^T^	HQ876043	U45776
*Candida argentea*	CBS 12358^T^	JF682350/	JF682353
*Candida baotianmanensis*	CBS 13915^T^	KM586743	KM586733
*Candida blackwellae*	CBS 10843^T^	EU402940/	EU402939
*Candida bohiensis*	NRRL Y-27737^T^	FJ172255	AY520317
*Candida buenavistaensis*	NRRL Y-27734^T^	FJ623627	AY242341
*Candida cetoniae*	CBS 12463 ^T^	KC118129	KC118128
*Candida chauliodes*	NRRL Y-27909 ^T^	FJ623621	DQ655678
*Candida coleopterorum*	CBS 14180 ^T^	KU128707	KU128722
*Candida corydalis*	NRRL Y-27910^T^	FJ623622	DQ655679
*Candida dubliniensis*	NRRL Y-17841^T^	KY102055	U57685
*Candida frijolesensis*	NRRL Y-48060^T^	EF658666	EF120596
*Candida hyderabadensis*	NRRL Y-27953^T^	AM180949	AM159100
*Candida jeffriesii*	CBS 9898 ^T^	NR_111398	NG_042498
*Candida jiufengensis*	CBS 10846 ^T^	EU402936	EU402935
*Candida kantuleensis*	CBS 15219^T^	LC317101	LC317097
*Candida labiduridarum*	NRRL Y-27940 ^T^	EF658664	DQ655687
*Candida lyxosophila*	NRRL Y-17539 ^T^	KY102184	HQ263370
*Candida maltosa*	NRRL Y-17677 ^T^	NR_138346	U45745
*Candida margitis*	CBS 14175 ^T^	KU128708	KU128721
*Candida materiae*	CBS 10975^T^	FJ154790	FJ154790
*Candida metapsilosis*	CBS 10907^T^	FJ872019	DQ213057
*Candida morakotiae*	NBRC 105009 ^T^	AB696987	DQ400364
*Candida neerlandica*	NRRL Y-27057^T^	EF658662	AF245404
*Candida oleophila*	NRRL Y-2317 **^T^**	AJ539374/	U45793
*Candida orthopsilosis*	ATCC MYA-96139^T^	FJ545241	DQ213056
*Candida oxycetoniae*	CBS 10844^T^	KY102281	EU402933
*Candida parablackwellae*	NYNU 17763^T^	MG255731	MG255702
*Candida parachauliodis*	CBS 13928 ^T^	KP054272	KP054271
*Candida parapsilosis*	NRRL Y-12969^T^	AJ635316	U45754
*Candida pseudojiufengensis*	CBS 10847 ^T^	EU402938	EU402937
*Candida pseudoviswanathii*	CBS 13916 ^T^	KM586736	KM586735
*Candida sanyaensis*	CBS 12637^T^	JQ647915	JQ647914
*Candida sakaeoensis*	CBS 12318 ^T^	AB696985	AB617978
*Candida sojae*	NRRL Y-17909^T^	KJ722419	U71070
*Candida subhashii*	CBS 10753 ^T^	NR_073356	EU836708
*Candida tetrigidarum*	NRRL Y-48142 ^T^	FJ623630	EF120599
*Candida theae*	ATCC MYA-4746^T^	JQ812707	JQ812701
*Candida tropicalis*	NRRL Y-12968^T^	AF287910	U45749
*Candida verbasci*	CBS 12699^T^	JX515982	JX515981
*Candida viswanathii*	CBS 4024^T^	KY102515	U45752
*Candida xiaguanensis*	CBS 13923^T^	KM586732	KM586731
*Candida yunnanensis*	NYNU 17948 ^T^	MG255721	MG255709
*Lodderomyces beijingensis*	CBS 14171 ^T^	KU128709	KU128720
*Lodderomyces elongisporus*	NRRL YB-4239 ^T^	AY391848	U45763
*Nematodospora anomalae*	CBS 13927^T^	KP054270	KP054269
*Nematodospora valgi*	CBS 12562 ^T^	KM386993	HM627112
*Scheffersomyces stipitis*	NRRL Y-7124^T^	JN943257/	U45741
*Spathaspora allomyrinae*	CBS 13924^T^	KP054268	KP054267
*Spathaspora arborariae*	ATCC MYA-4684^T^	NR_111592	NG_042574
*Spathaspora boniae*	CBS 13262^T^	NR_158910	KT276332
*Spathaspora brasiliensis*	CBS 12679 ^T^	JN099271	JN099271
***Spathaspora elongata***	**NYNU 18115^T^**	**MK682770**	**MK682796**
***Spathaspora elongata***	**NYNU 181112**	**MT276033**	**MT274662**
***Spathaspora elongata***	**NYNU 181120**	**MT276034**	**MT276036**
***Spathaspora elongata***	**NYNU 181158**	**MT276035**	**MT276032**
*Spathaspora girioi*	CBS 13476^T^	NR_155783	NG_059955
*Spathaspora gorwiae*	CBS 13472 ^T^	NR_155784	NG_059956
*Spathaspora hagerdaliae*	CBS 13475^T^	NR_155800	KU556168
***Spathaspora jiuxiensis***	**NYNU 17416 ^T^**	**MG255706**	**MG255718**
***Spathaspora jiuxiensis***	**NYNU 17417**	**MT276035**	**MT276032**
***Spathaspora mengyangensis***	**NYNU 17741^T^**	**KY213816**	**KY213819**
***Spathaspora mengyangensis***	**NYNU 17705**	**MT272353**	**MT272351**
***Spathaspora parajiuxiensis***	**NYNU 16747 ^T^**	**MG255728**	**MG255705**
***Spathaspora parajiuxiensis***	**NYNU 16632**	**MT272352**	**MT272350**
*Spathaspora passalidarum*	NRRL Y-27907^T^	NR_111397	DQ109807
*Spathaspora piracicabensis*	CBS 15054^T^	KR864907	KR864906
***Spathaspora rosae***	**NYNU 17934^T^**	**MG255725**	**MG255701**
***Spathaspora rosae***	**NYNU 17903**	**MT274659**	**MT274661**
***Spathaspora rosae***	**NYNU 17909**	**MT274664**	**MT274663**
*Spathaspora roraimanensis*	CBS 12681^T^	JN099269	JN099269
*Spathaspora suhii*	CBS 12680^T^	JN099270	JN099270
*Spathaspora xylofermentans*	CBS 12682^T^	JN099268	JN099268
*Wickerhamia fluorescens*	JCM 1821**^T^**	NR_111311/	NG_054831

Abbreviations: **ATCC**: American Type Culture Collection, Manassas, VA, USA; **CBS**: CBS-KNAW Collections, Westerdijk Fungal Biodiversity Institute, Utrecht, The Netherlands; **JCM**: RIKEN BioResource Research Center-Japan Collection of Microorganisms, Takao, Japan; **NRRL**: the ARS Culture Collection, National Center for Agricultural Utilization Research, Peoria, IL, USA; **NYNU**: Microbiology Lab, Nanyang Normal University, Henan, China; **T**: type strain.

## Results

### Phylogenetic analyses

**Figure 1.**
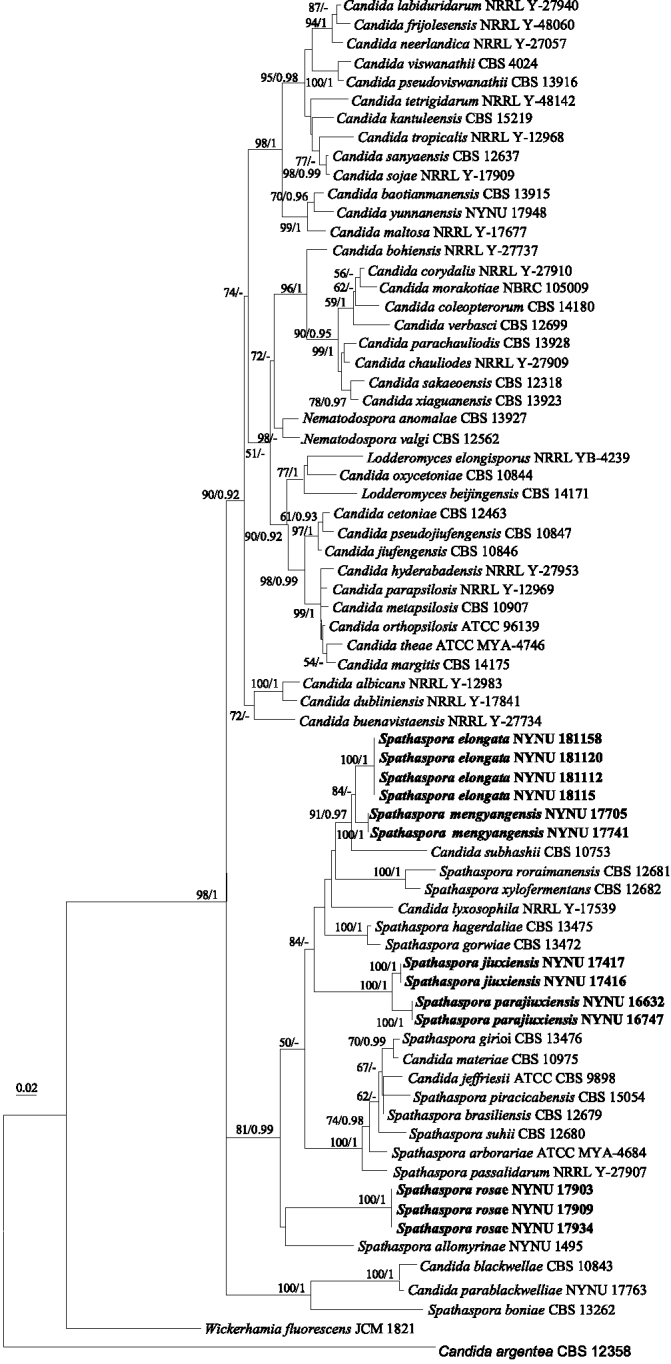
Phylogenetic tree, based on an ML analysis of a combined DNA dataset of ITS and nuc 28S rDNA sequences for *Spathaspora* species and related taxa in the Debaryomycetaceae. Numbers above the branches indicate ML bootstraps (left, MLBS ≥ 50%) and Bayesian Posterior Probabilities (right, BPP ≥ 0.90). The tree is rooted with sequences from *Candida argentea*CBS 12358. Isolates from the current study are shown in bold letters. “-” indicates MLBS < 50% or BPP < 0.90. The scale bar indicates the number of substitutions per site.

The combined nuclear dataset (ITS and nuc 28S) was analysed to infer the interspecific relationships within the larger *Spathaspora*/*Candida albicans*/*Lodderomyces* clade of Debaryomycetaceae. The dataset consisted of 72 sequences including the outgroup, *Candida argentea* (culture CBS 12358). A total of 944 characters including gaps (391 for ITS and 553 for nuc 28S) were included in the phylogenetic analysis. The best nucleotide substitution model for ITS and nuc 28S was GTR+I+G. ML and BI analyses of the combined dataset resulted in phylogenetic reconstructions with similar topologies and the average standard deviation of split frequencies was 0.011210 (BI). In the ML phylogenetic tree (Figure 1), thirteen strains formed five single clades with high to full support (100% in ML and 0.99 or 1.00 in BI) and clustered in the clade that comprised most species of *Spathaspora*. Phylogenetically, *S. elongata* and *S. mengyangensis* clustered together with high support (84% in ML and 0.91 in BI), while *S. jiuxiensis* and *S. parajiuxiensis* clustered together with strong support (100% in ML and 1.00 in BI). Two strains of *S. rosae* formed a unique lineage with *S. allomyrinae*, but with low support.

### Taxonomy

#### Spathaspora elongata

Taxon classificationFungiSaccharomycetalesDebaryomycetaceae

C.Y. Chai & F.L. Hui sp. nov.

3FC25792-55DD-5C84-96FC-B8FFA2876FD7

836444

Figure 2

##### Type.

China, Yunnan Province, Jinghong City, Mengyang Town, in rotting wood from a tropical rainforest, August 2018, K.F. Liu & Z.W. Xi (holotype, NYNU 18115^T^ preserved in a metabolically-inactive state), ex-holotype: CICC 33353; CBS 16002.

##### Etymology.

*Elongata* refers to the elongate ascospores of this yeast.

##### Description.

**Figure 2.**
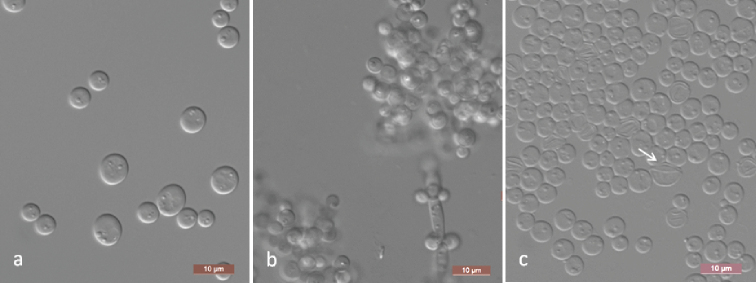
Morphology of *Spathaspora elongata* (NYNU 18115, holotype) **a** budding cells on YM broth after 3 d **b** Pseudohyphae on CM agar after 14 d **c** ascus and ascospore (arrow) on dilute V8 agar after 14 d. Scale bars: 10 μm.

After 3 days of culture in YM broth at 25 °C, the cells are ovoid (3–4 × 3–7 μm) and occur singly or in pairs (Fig. 2a). Budding is multilateral. Sediment is formed after a month, but a pellicle is not observed. After 3 days of growth on YM agar at 25 °C, colonies are white to cream-coloured, butyrous and smooth with entire margins. After 14 days at 25 °C, on Dalmau plate culture on CM agar, pseudohyphae are present, but true hyphae are not formed (Fig. 2b). Sporulation occurs on dilute (1:19) V8 agar after 14 days at 25 °C. Unconjugated asci are formed from single cells with one elongated ascospore which are tapered and curved at the ends (Fig. 2c). Glucose, galactose, maltose and sucrose are weakly fermented. Xylose fermentation is absent using Durham tubes, but ethanol is produced from xylose when determined with alcohol oxidase and peroxidase tests. Glucose, d-ribose, d-xylose, d-arabinose, sucrose, maltose, trehalose, methyl α-d-glucoside, cellobiose, salicin, arbutin, inulin, ribitol, d-glucitol, d-mannitol, 2-keto-d-gluconate, succinate, citrate and ethanol are assimilated. No growth occurs with galactose, l-sorbose, d-glucosamine, l-arabinose, l-rhamnose, melibiose, lactose, raffinose, melezitose, glycerol, erythritol, xylitol, galactitol, *myo*-inositol, d-glucono-1, 5-lactone, 5-keto-d-gluconate, d-gluconate, d-glucuronate, dl-lactate or methanol. For the assimilation of nitrogen compounds, growth on ethylamine, l-lysine, glucosamine or d-tryptophan is present, whereas growth on nitrate, nitrite, cadaverine, creatine, creatinine or imidazole is absent. Growth is observed at 37 °C but not at 40 °C. Growth in the presence of 1% acetic acid is present, but growth in the presence of 10% sodium chloride (NaCl) plus 5% glucose and 0.01% cycloheximide is absent. Starch-like compounds are not produced. Urease activity and diazonium blue B reactions are negative.

##### Additional isolates examined.

China, Yunnan Province, Jinghong City, Mengyang Town, in rotting wood from a tropical rainforest, August 2018, K.F. Liu & Z.W. Xi, NYNU 181112, NYNU 181120 and NYNU 181158.

##### Notes.

Four strains, representing *Sp. elongata*, clustered in a well-supported phylogenetic clade that is closely related to *Sp. mengyangensis*, another new species proposed in this paper and *C. subhashii*. The nucleotide differences between *Sp. elongata* and *Sp. mengyangensis* were 2.5% substitutions in the D1/D2 domain and 5.2% substitutions in the ITS region (Groenewald et al. 2016). Similarly, *Sp. elongata* and *C. subhashii* showed differences of 3.9% substitutions in the D1/D2 domain and 5.9% substitutions in the ITS region (Groenewald et al. 2016). Physiologically, *Sp. elongata* can be differentiated from its close relative, *Sp. mengyangensis*, based on its growth in citrate and the presence of 1% acetic acid, which are present for *Sp. elongata* and absent for *Sp. mengyangensis*. Moreover, *Sp. elongata* weakly ferments glucose, galactose, maltose and sucrose and grows at 37 °C, but *Sp. mengyangensis* does not.

#### Spathaspora mengyangensis

Taxon classificationFungiSaccharomycetalesDebaryomycetaceae

C.Y. Chai & F.L. Hui sp. nov.

4FA77618-533E-5262-A5E3-F8DED137CA00

836445

Figure 3

##### Type.

China, Yunnan Province, Jinghong City, Mengyang Town, in rotting wood from a tropical rainforest, July 2017, K.F. Liu & L. Zhang (holotype, NYNU 17741^T^ preserved in a metabolically-inactive state), ex-holotype: CICC 33267; CBS 15227.

##### Etymology.

*Mengyangensis* refers to the geographical origin of the type strain of this species.

##### Description.

**Figure 3.**
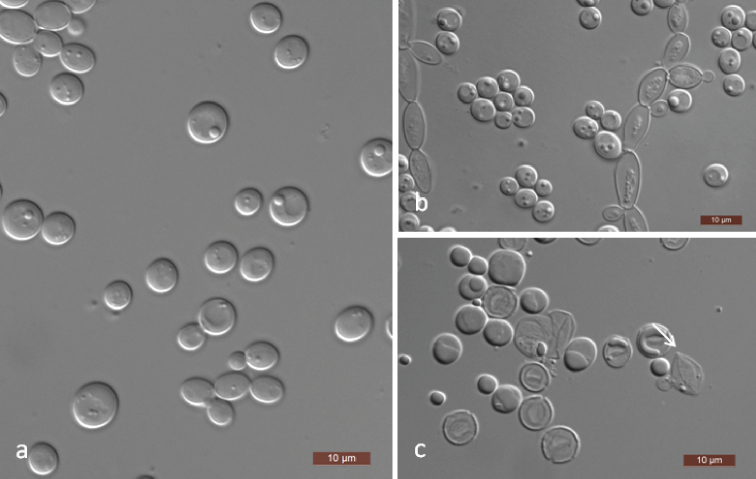
*Spathaspora mengyangensis* (NYNU 17741, holotype) **a** budding cells on YM broth after 3 d **b** simple pseudohyphae on CM agar after 14 d **c** ascus and ascospore (arrow) on CM agar after 14 d. Scale bars: 10 μm.

In YM broth after 3 days at 25 °C, cells are ovoid (3–7 × 5–7.5 μm) and occur singly or in pairs (Fig. 3a). Budding is multilateral. Sediment is formed after a month, but a pellicle is not observed. After 3 days of growth on YM agar at 25 °C, colonies are white to cream-coloured, butyrous and smooth with entire margins. After 14 days at 25 °C on Dalmau plate culture on CM agar, pseudohyphae are present, but true hyphae are not formed (Fig. 3b). Sporulation occurs on CM agar after 14 days at 25 °C. Unconjugated asci are formed from single cells with one elongated ascospore which are tapered and curved at the ends (Fig. 3c). Xylose fermentation is negative using Durham tubes, but ethanol is produced from xylose when determined with alcohol oxidase and peroxidase tests. Glucose, d-ribose, d-xylose, sucrose, maltose, trehalose, methyl α-d-glucoside, cellobiose, salicin, arbutin, inulin, ribitol, d-glucitol, d-mannitol, 2-keto-d-gluconate, succinate and ethanol are assimilated. No growth occurs with galactose, l-sorbose, d-glucosamine, l-arabinose, d-arabinose, l-rhamnose, melibiose, lactose, raffinose, melezitose, glycerol, erythritol, xylitol, galactitol, *myo*-inositol, d-glucono-1, 5-lactone, 5-keto-d-gluconate, d-gluconate, d-glucuronate, dl-lactate, citrate or methanol. For the assimilation of nitrogen compounds, growth on ethylamine, l-lysine, glucosamine or d-tryptophan is present, whereas growth on nitrate, nitrite, cadaverine, creatine, creatinine or imidazole is absent. Growth is observed at 30 °C, but not at 35 °C. Growth in the presence of 10% NaCl plus 5% glucose, 0.01% cycloheximide and 1% acetic acid is absent. Starch-like compounds are not produced. Urease activity and diazonium blue B reactions are negative.

##### Additional isolate examined.

China, Yunnan Province, Jinghong City, Mengyang Town, in rotting wood from a tropical rainforest, July 2017, K.F. Liu & L. Zhang, NYNU 17705.

##### Notes.

Phylogenetic analyses show that *Sp. mengyangensis* is closely related to *Sp. elongata* and *C. subhashii*; however, the independent phylogenetic position and different physiological characters can distinguish *Sp. mengyangensis* from its sister species *Sp. elongata* (as mentioned above). Similarly, *Sp. mengyangensis* differed from *C. subhashii* by 2.8% substitutions in the D1/D2 domain and 7.8% substitutions in the ITS region (Groenewald et al. 2016). Physiologically, *Sp. mengyangensis* can be differentiated from *C. subhashii* by the ability to assimilate d-ribose, trehalose, d-glucitol and d-mannitol and the inability to assimilate galactose, l-arabinose and melezitose. In addition, *C. subhashii* can grow at 40 °C, but *Sp. mengyangensis* cannot.

#### Spathaspora jiuxiensis

Taxon classificationFungiSaccharomycetalesDebaryomycetaceae

C.Y. Chai & F.L. Hui sp. nov.

749F6068-3E6A-58F6-801B-77710D2D60A9

836446

Figure 4

##### Type.

China, Yunnan Province, Honghe Prefecture, Luxi County, in rotting wood in Jiuxi Mountain Forest Park, July 2017, K.F. Liu & L. Zhang (holotype, NYNU 17416^T^ preserved in a metabolically-inactive state), ex-holotype: CICC 33264; CBS 15226.

##### Etymology.

*Jiuxiensis* refers to Jiuxi Mountain, the mountain from which it was collected.

##### Description.

**Figure 4.**
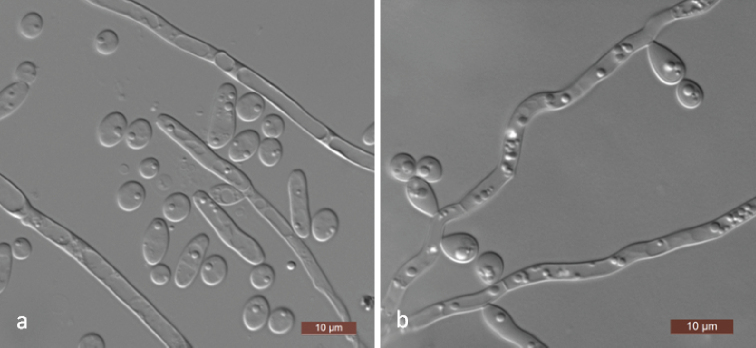
Morphology of *Spathaspora jiuxiensis* (NYNU 17416, holotype) **a** budding cells and pseudohyphae on YM broth after 3 d **b** true hyphae with blastoconidia on CM agar after 14 d. Scale bars: 10 μm.

In YM broth after 3 days at 25 °C, cells are ovoid to elongate (3–6 × 3.5–9 μm) and occur singly or in pairs (Fig. 4a); pseudohyphae are present. Budding is multilateral. Sediment is formed after a month, but a pellicle is not observed. After 3 days of growth on YM agar at 25 °C, colonies are white to cream-coloured, butyrous and smooth with entire margins. After 12 days at 25 °C on Dalmau plate culture on CM agar, pseudohyphae and true hyphae are formed (Fig. 4b). Asci or signs of conjugation were not seen on the sporulation media used. Glucose and maltose are weakly fermented. Xylose fermentation is negative using Durham tubes, but ethanol is produced from xylose when determined with alcohol oxidase and peroxidase tests. Glucose, d-glucosamine, d-ribose, d-xylose, sucrose, maltose, trehalose, methyl α-d-glucoside, cellobiose, salicin, arbutin, melezitose, inulin, ribitol, d-glucitol, d-mannitol, 2-keto-d-gluconate, dl-lactate, succinate and ethanol are assimilated. No growth occurs with galactose, l-sorbose, l-arabinose, d-arabinose, l-rhamnose, melibiose, lactose, raffinose, glycerol, erythritol, xylitol, galactitol, *myo*-inositol, d-glucono-1, 5-lactone, 5-keto-d-gluconate, d-gluconate, d-glucuronate, citrate, l-arabinitol or methanol. For the assimilation of nitrogen compounds, growth on l-lysine, glucosamine or d-tryptophan is present, whereas growth on nitrate, nitrite, ethylamine, cadaverine, creatine, creatinine or imidazole is absent. Growth is observed at 35 °C, but not at 37 °C. Growth in the presence of 0.01% cycloheximide is present, but growth in the presence of 0.1% cycloheximide, 10% NaCl plus 5% glucose and 1% acetic acid is absent. Starch-like compounds are not produced. Urease activity and diazonium blue B reactions are negative.

##### Additional isolate examined.

China, Yunnan Province, Honghe Prefecture, Luxi County, in rotting wood in Jiuxi Mountain Forest Park, July 2017, K.F. Liu & L. Zhang, NYNU 17417.

##### Notes.

The two strains, both representing *Sp. jiuxiensis*, cluster in a well-supported clade in the phylogenetic analysis and are closely related to *Sp. parajiuxiensis*. The nucleotide differences between these two new species were 1.4% substitutions in the D1/D2 domain and 4.6% substitutions in the ITS region (Groenewald et al. 2016). These two sister species can also be differentiated by a few physiological characteristics; *Sp. jiuxiensis* can assimilate dl-lactate and *Sp. parajiuxiensis* can grow at 37 °C.

#### Spathaspora parajiuxiensis

Taxon classificationFungiSaccharomycetalesDebaryomycetaceae

C.Y. Chai & F.L. Hui sp. nov.

B22E2D63-311B-5F24-88AF-69FFDE522A0F

836447 Figure 5

##### Type.

China, Yunnan Province, Honghe Prefecture, Luxi County, in rotting wood in Jiuxi Mountain Forest Park, July 2016, R.C. Ren & L. Zhang (holotype, NYNU 16747^T^ preserved in a metabolically-inactive state), ex-holotype: CICC 33162; CBS 14691.

##### Etymology.

*Paraluxiensis* refers to its close phylogenetic relationship to *Sp. luxiensis*.

##### Description.

**Figure 5.**
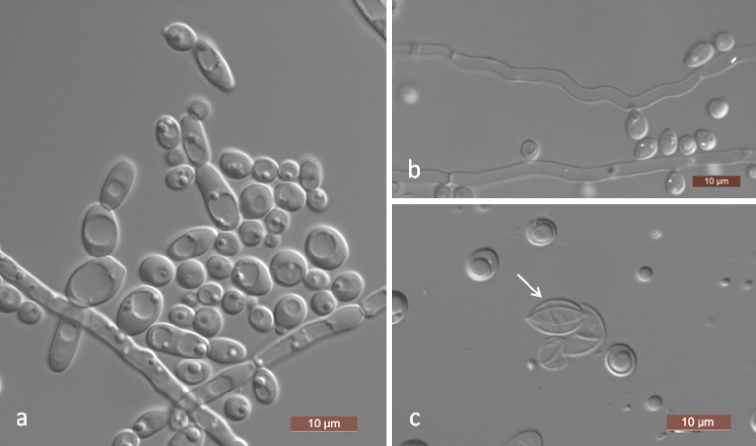
Morphology of *Spathaspora parajiuxiensis* (NYNU 16747, holotype) **a** budding cells and pseudohyphae on YM broth after 3 d **b** true hyphae with blastoconidia on CM agar after 14 d **c** ascus and ascospore (arrow) on 5% ME agar after 14 d. Scale bars: 10 μm.

In YM broth after 3 days at 25 °C, cells are ovoid to elongate (3.5–4 × 7–15 μm) and occur singly or in pairs (Fig. 5a); pseudohyphae are present. Budding is multilateral. Sediment is formed after a month, but a pellicle is not observed. After 3 days of growth on YM agar at 25 °C, colonies are white to cream-coloured, butyrous and smooth with entire margins. After 12 days at 25 °C on Dalmau plate culture on CM agar, pseudohyphae and true hyphae are formed (Fig. 5b). Sporulation occurs on 5% ME agar after 14 days at 25 °C. Unconjugated asci are formed from single cells with one elongated ascospores which are tapered and curved at the ends (Fig. 5c) Glucose and maltose are weakly fermented. Xylose fermentation is negative using Durham tubes, but ethanol is produced from xylose when determined with alcohol oxidase and peroxidase tests. Glucose, d-glucosamine, d-ribose, d-xylose, sucrose, maltose, trehalose, methyl α-d-glucoside, cellobiose, salicin, arbutin, melezitose, inulin, ribitol, d-glucitol, d-mannitol, 2-keto-d-gluconate, succinate and ethanol are assimilated. No growth occurs with galactose, l-sorbose, l-arabinose, d-arabinose, l-rhamnose, melibiose, lactose, raffinose, glycerol, erythritol, xylitol, galactitol, *myo*-inositol, d-glucono-1, 5-lactone, 5-keto-d-gluconate, d-gluconate, d-glucuronate, dl-lactate, citrate, l-arabinitol or methanol. For the assimilation of nitrogen compounds, growth on l-lysine, glucosamine or d-tryptophan is present, whereas growth on nitrate, nitrite, ethylamine, cadaverine, creatine, creatinine or imidazole is absent. Growth is observed at 37 °C, but not at 40 °C. Growth in the presence of 0.01% cycloheximide is present, but growth in the presence of 0.1% cycloheximide, 10% NaCl plus 5% glucose and 1% acetic acid is absent. Starch-like compounds are not produced. Urease activity and diazonium blue B reactions are negative.

##### Additional isolate examined.

China, Yunnan Province, Honghe Prefecture, Luxi County, in rotting wood in Jiuxi Mountain Forest Park, July 2016, R.C. Ren & L. Zhang, NYNU 16632.

#### Spathaspora rosae

Taxon classificationFungiSaccharomycetalesDebaryomycetaceae

C.Y. Chai & F.L. Hui sp. nov.

F19D533C-9119-5360-BC7D-612C74AB01B9

836448

Figure 6

##### Type.

China, Yunnan Province, Jinghong City, Mengyang Town, in rotting wood in a tropical rainforest, July 2017, Z.W. Xi & L. Zhang (holotype, NYNU 17934^T^ preserved in a metabolically-inactive state), ex-holotype: CICC 33271; CBS 15231.

##### Etymology.

*Rosae* was named in honour of Carlos A. Rosa for his contributions in yeast taxonomy.

##### Description.

**Figure 6.**
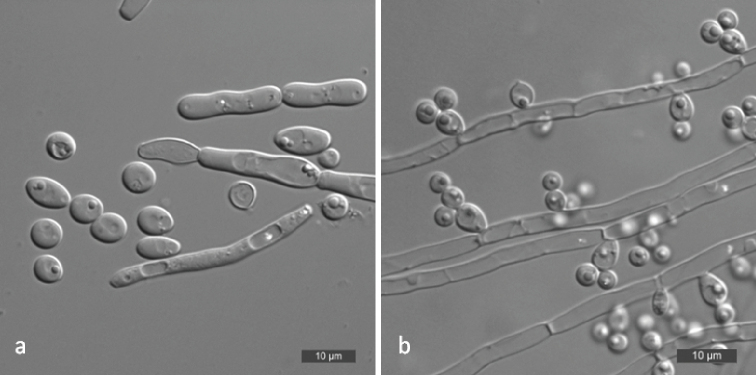
Morphology of *Spathaspora rosae* (NYNU 17934, holotype) **a** budding cells and elongated vegetative cells on YM broth after 3 d **b** true hyphae with blastoconidia on CM agar after 14 d. Scale bars: 10 μm.

In YM broth after 3 days at 25 °C, cells are ovoid to elongate (4–7 × 5–16 μm) and occur singly or in pairs (Fig. 6a). Budding is multilateral. Sediment is formed after a month, but a pellicle is not observed. After 3 days of growth on YM agar at 25 °C, colonies are white to cream-coloured, butyrous and smooth with entire margins. After 7 days at 25 °C, on Dalmau plate culture on CM agar, pseudohyphae and true hyphae are formed (Fig. 6b). Asci or signs of conjugation are not seen on sporulation media used. Xylose fermentation is negative using Durham tubes, but ethanol is produced from xylose when determined with alcohol oxidase and peroxidase tests. Glucose, d-glucosamine, d-xylose, sucrose, maltose, trehalose, methyl α-d-glucoside, cellobiose, salicin, arbutin, inulin, ribitol, d-glucitol, d-mannitol, 2-keto-d-gluconate, dl-lactate, succinate, citrate and ethanol are assimilated. No growth occurs with galactose, l-sorbose, d-ribose, l-arabinose, d-arabinose, l-rhamnose, melibiose, lactose, raffinose, melezitose, glycerol, erythritol, xylitol, galactitol, *myo*-inositol, d-glucono-1, 5-lactone, 5-keto-d-gluconate, d-gluconate, d-glucuronate, l-arabinitol or methanol. For the assimilation of nitrogen compounds, growth on ethylamine, l-lysine, glucosamine or d-tryptophan is present, whereas growth on nitrate, nitrite, cadaverine, creatine, creatinine or imidazole is absent. Growth is observed at 35 °C, but not at 37 °C. Growth in the presence of 0.01% cycloheximide is present, but growth in the presence of 0.1% cycloheximide, 10% NaCl plus 5% glucose and 1% acetic acid is absent. Starch-like compounds are not produced. Urease activity and diazonium blue B reactions are negative.

##### Additional isolates examined.

China, Yunnan Province, Jinghong City, Mengyang Town, in rotting wood in a tropical rainforest, July 2017, Z.W. Xi & L. Zhang NYNU 17903, NYNU 17909.

##### Notes.

Three strains, representing *Sp. rosae*, grouped in a well-supported clade and appear to be most closely related to *Sp. allomyrinae* (Wang et al. 2016). The nucleotide differences between *Sp. rosae* and its close relative, *Sp. allomyrinae*, were 10.2% substitutions in the D1/D2 domain and 11% substitutions in the ITS region (Groenewald et al. 2016). Physiologically, *Sp. rosae* can be differentiated from *Sp. allomyrinae*, based on growth in galactose, melezitose, xylitol and 5-keto-d-gluconate, which are positive for *Sp. allomyrinae* and negative for *Sp. rosae*. Moreover, *Sp. allomyrinae* weakly ferments glucose, galactose, maltose and cellobiose, but *Sp. rosae* does not.

### Two new combinations

In addition to the previously-described taxa, two new combinations are proposed herein and their descriptions refer to relevant protologues.

#### Spathaspora materiae

Taxon classificationFungiSaccharomycetalesDebaryomycetaceae

(Barbosa, Cadete, Gomes, Lachance & Rosa) C.Y. Chai & F.L. Hui comb. nov.

EFD267D3-5CDB-578E-B858-7609C62CF0E7

##### Basionym.

*Candida materiae* Barbosa, Cadete, Gomes, Lachance & Rosa, International Journal of Systematic and Evolutionary Microbiology 59(8): 2015 (2009).

#### Spathaspora jeffriesii

Taxon classificationFungiSaccharomycetalesDebaryomycetaceae

(N.H. Nguyen, S.-O. Suh & M. Blackwell) C.Y. Chai & F.L. Hui comb. nov.

3618ABA1-5493-59C1-97E5-DCCAE8EBE5E3

##### Basionym.

*Candida jeffriesii* N.H. Nguyen, S.-O. Suh & M. Blackwell, Mycological Research 110(10): 1239 (2006).

## Discussion

*Spathaspora* is distributed worldwide with 12 species identified from rotting wood and insects. In China, three species of *Spathaspora* have been previously reported (Ren et al. 2013; Wang et al. 2016). In the present study, five additional species, *Sp. elongata*, *Sp. jiuxiensis*, *Sp. mengyangensis*, *Sp. parajiuxiensis* and *Sp. rosae* (Fig. 1), were recorded in addition to previously-known species. Thus, to our knowledge, eight species of *Spathaspora* are currently known from China. Of these eight species, only two species, *Sp. gorwiae* and *Sp. passalidarum*, were reported in China up until 2013 (Ren et al. 2013). The remaining six species, namely *Sp. allomyrinae*, *Sp. elongata*, *Sp. jiuxiensis*, *Sp. mengyangensis*, *Sp. parajiuxiensis* and *Sp. rosae*, were recorded from 2016 to 2018. Given this history, it is most likely that more species will be found. Nonetheless, this number is significant when compared to the total diversity of 11 species of *Spathaspora* reported for South America (Cadete et al. 2009, 2013; Lopes et al. 2016; Morais et al. 2017; Varize et al. 2018). Further studies are needed to document the overall diversity of species of *Spathaspora* in China, especially in the southwest regions.

The phylogenetic relationship of *Spathaspora* has been unclear until now, mainly due to its polyphyletic nature (Daniel et al. 2014; Morais et al. 2017; Varize et al. 2018). In this article, we used more available type strains to revise this genus, based on a phylogenetic analysis of the combined ITS and nuc 28S rDNA sequences. As shown in Figure 1, three main groups were reconstructed and the results showed that *Spathaspora* is not a monophyletic group, but rather is polyphyletic with several *Candida* species included. *Sp. passalidarum*, the type species of the genus, *C. jeffriesii*, *C. materiae*, *Sp. arborariae*, *Sp. brasiliensis*, *Sp. girioi* and *Sp. suhii* form a core group that is well supported by phylogeny. This result is similar to the results of previous phylogenetic analyses of nuc 28S rDNA sequences (Morais et al. 2017; Varize et al. 2018). Therefore, two *Candida* species, *C. materiae* and *C. jeffriesii*, are transferred to *Spathaspora* as new combinations because of their phylogenetic placement within that genus.

The second group is composed of ten distinct species, including the four species *Sp. elongata*, *Sp. mengyangensis*, *Sp. jiuxiensis* and *Sp. parajiuxiensis* described in this study. Typical ascospores are formed by *Sp. elongata*, *Sp. mengyangensis*, *Sp. parajiuxiensis* and *Sp roraimanensis*, but other members of the group are known from their asexual cycle only.

The species *Sp. allomyrinae*, which shares the unique ascospore morphology of the genus, fell outside a larger *Spathaspora* clade, as in the nuc 28S-based phylogeny proposed by Morais et al. (2017). However, this species is joined by *Sp. rosae*, which is described in the current study, in a third cluster consisting of *Spathaspora* in our phylogenetic analysis (Fig. 1). Placement of *Sp. allomyrinae* and *Sp. rosae* is only weakly supported and continued assignment to the genus will require verification from more robust datasets, such as whole genome sequences.

Morais et al. (2017) described the species *Sp. boniae*, based on two strains producing asci containing elongate ascospores with curved ends typical of the genus *Spathaspora*. Our phylogenomic analysis showed that *Sp. boniae* clusters with *C. blackwellae* and *C. parablackwellae* to form a distinct clade outside a larger *Spathaspora* clade. This result was also supported by previous phylogenetic analyses on this clade using nuc 28S rDNA sequences (Morais et al. 2017; Varize et al. 2018; Zhai et al. 2019). These results suggest that the genus *Spathaspora* should be limited to species in the group comprising the type species *Sp. passalidarum*. This clade, which has been treated previously as members of *Spathaspora*, may represent a separate genus, despite the morphological characteristics of the included species and isolates are similar to *Spathaspora*. Therefore, whole genome sequencing of all *Spathaspora* species and those of related genera, combined with the discovery of new species of the clade, is needed to clarify the possible heterogeneity of this genus.

*Spathaspora* is a cosmopolitan genus, but most known species have relatively-distinct habitats or regional locations. Currently, most *Spathaspora* species are known from East Asia (mainly in China) and South America. Although the taxonomy of *Spathaspora* has received much attention in recent years, many regions in the world are under-sampled and more under-described indigenous *Spathaspora* species will undoubtedly be discovered in the future as with most microfungal genera (Hyde et al. 2020). Our study indicates that there is a high species diversity of *Spathaspora* waiting to be discovered in rotting wood in tropical and subtropical southwest China and nearby areas as with other genera (Hyde et al. 2018).

## Supplementary Material

XML Treatment for Spathaspora elongata

XML Treatment for Spathaspora mengyangensis

XML Treatment for Spathaspora jiuxiensis

XML Treatment for Spathaspora parajiuxiensis

XML Treatment for Spathaspora rosae

XML Treatment for Spathaspora materiae

XML Treatment for Spathaspora jeffriesii
